# Unraveling Redox Mediator‐Assisted Chemical Relithiation Mechanism for Direct Recycling of Spent Ni‐Rich Layered Cathode Materials

**DOI:** 10.1002/advs.202417094

**Published:** 2025-01-22

**Authors:** Suji Kim, Ukseon Shin, Hyun Ju Yoon, Soo‐Ah Yoon, Jinju Song, Jiyoung Ma, Jung‐Je Woo, Kyung‐Wan Nam, Dong‐Hwa Seo, Won‐Hee Ryu

**Affiliations:** ^1^ Department of Chemical and Biological Engineering Sookmyung Women's University 100 Cheongpa‐ro 47‐gil, Yongsan‐gu Seoul 04310 Republic of Korea; ^2^ Department of Materials Science and Engineering Korea Advanced Institute of Science and Technology (KAIST) 291 Daehak‐ro Daejeon 34141 Republic of Korea; ^3^ Department of Energy & Materials Engineering Dongguk University Seoul 04620 Republic of Korea; ^4^ Gwangju Clean Energy Research Center Korea Institute of Energy Research (KIER) 270‐25 Samso‐ro Gwangju 61003 Republic of Korea

**Keywords:** cathode, chemical relithiation, direct recycling, Li‐ion batteries, redox‐mediator

## Abstract

The increasing demand for Li‐ion batteries across various energy storage applications underscores the urgent need for environmentally friendly and efficient direct recycling strategies to address the issue of substantial cathode waste. Diverse reducing agents for Li supplements, such as quinone molecules, have been considered to homogenize the Li distribution in the cathode materials obtained after cycling; however, the detailed reaction mechanism is still unknown. Herein, the ideal electrochemical potential factor and reaction mechanism of the redox mediator 3,5‐di‐tert‐butyl‐o‐benzoquinone (DTBQ) for the chemical relithiation of high‐Ni‐layered cathodes are elucidated. Here, 100% efficiency of DTBQ‐assisted chemical relithiation is achieved by adjusting the direct immersion time of Li‐deficient cathode electrodes. The reversible reaction features of the physical and chemical structures of both the regenerated cathodes and the DTBQ molecules are investigated using advanced characterization and density functional theory calculations. These findings emphasize the potential of redox‐mediator‐assisted chemical relithiation for realizing direct recycling processes and offer a facile and sustainable solution for battery recycling.

## Graphical abstract

3,5‐di‐tert‐butyl‐o‐benzoquinone (DTBQ) promotes the chemical relithiation of Li‐deficient NCM622 cathodes through a redox reaction, achieving 100% efficiency in Li replenishment. Chemical relithiation effectively restores the structural and electrochemical properties of cathodes, thereby enhancing their electrochemical performance and cycle stability. This approach provides a feasible and environmentally friendly recycling method for Li‐ion batteries.

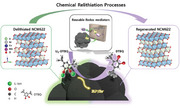

## 1 Introduction

Lithium‐ion batteries (LIBs) satisfy the exceptional demand for energy storage, and have a wide range of applications from small portable electronics to larger electric vehicles.^[^ ^1^ ^]^ The growing demand for LIBs has led to increasing concerns about the supply and cost risks associated with raw materials and the environmental impact of the proper disposal and effective treatment of spent LIBs.^[^ ^2^ ^]^ The improper disposal of spent LIBs results in the release of flammable and hazardous waste, including electrolytes and transition metals, posing significant risks of contamination to soil, water, and air.^[^ ^3^ ^]^ Therefore, sustainable and efficient recycling processes for spent LIB components, such as expensive cathode materials, need to be developed to address the environmental, economic, and supply challenges associated with raw material extraction and treatment.^[^ ^4^ ^]^

Three representative recycling methods for spent LIBs are considered: pyrometallurgy, hydrometallurgy, and direct recycling.^[^ ^5^ ^]^ Although pyrometallurgy and hydrometallurgy have been used to extract useful cathode elements, such as lithium and transition metals, these processes often suffer from unfavorable energy inputs (e.g., high temperature) and environmental concerns (hazardous liquid waste).^[^ ^6^ ^]^ To avoid the energy‐intensive and environmentally detrimental steps of conventional recycling processes, a direct recycling pathway is a sustainable alternative that aims to recover and reuse cathode materials with minimal performance degradation.^[^ ^7^ ^]^ The direct recycling method enables the preservation of the structure and composition of cathode materials via a nondestructive pathway, unlike pyrometallurgical or hydrometallurgical processes.^[^ ^8^ ^]^ This necessity arises not only from the objective of minimizing the environmental detriments associated with LIB waste, but also from the crucial objective of recovering vital elements in cathode materials, such as Li, Co, and Ni.^[^ ^9^ ^]^

To assure the quality and reliability of the cathode materials collected from direct recycling, it is crucial to address the non‐uniform lithium distribution among the repeatedly reacted cathode particles. Considerable efforts have been directed toward regenerating spent cathodes by adding Li sources via chemical relithiation. Chemical relithiation involves a redox reaction in which Li ions and electrons are transferred from a lithiation agent to a Li‐deficient cathode material.^[^ ^10^ ^]^ Typically, this is achieved by employing Li‐containing compounds, such as lithium salts or organolithium reagents, which react directly with the active material in the cathode.^[^ ^11^ ^]^ For instance, the introduction of a lithiated intermediary (e.g., a polycyclic aryl Li compound) facilitates concerted Li and electron transfer to the active sites of the cathode.^[^ ^12^ ^]^ This method is particularly notable for its efficiency and dramatically reduces processing time compared with other recycling methods. However, structural damage or crystal collapse is often observed within the cathode materials because of the strong reducing ability of certain aryl lithium reagents. Unexpectedly, these reagents cause overlithiation, leading to irreversible damage to the crystal structure of the cathode.

Alternatively, the introduction of redox mediators (RMs), such as quinone‐based and organic electron‐donating materials, has proven effective in the restoration and regeneration of layered Li[Ni_x_Co_y_Mn_z_]O_2_ (NCM, x + y + z = 1) cathode materials.^[^ ^13^ ^]^ The RM can rapidly transfer charges between the cathode and the Li metal, effectively delivering electrons and Li^+^ ions to relithiate the degraded cathode materials, thus offering a unique and efficient method for achieving a homogenous distribution of Li contents among all cathode particles.^[^ ^14^ ^]^ The Li replenishment process automatically terminates once the Li content reaches a specific value owing to the chemical potential. This eliminates the need to account for the degree of electrode degradation or the amount of lithium required for supplementation. According to recent studies, quinone‐based materials that act as RMs effectively replenish Li in Li‐deficient cathodes through chemical relithiation. Nevertheless, the detailed mechanisms of the RM in the lithiation process remain largely unknown. The Li replenishment process of cathode materials and specific interactions involving RMs have not yet been fully explored. Therefore, a detailed mechanistic study is required along with the development of effective chemical relithiation strategies using RMs to homogenize the Li content distribution among used cathode particles with consistent chemical potential levels.

**Figure 1:**
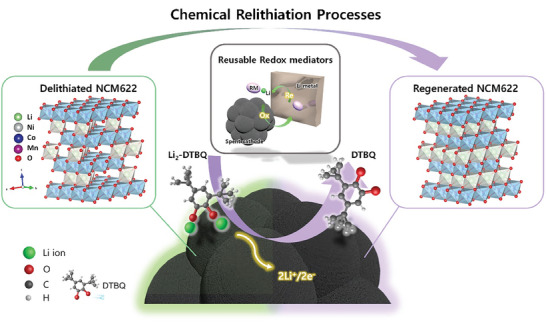
Schematic illustration of direct regeneration process via the DTBQ‐assisted chemical relithiation strategy of delithiated NCM622 materials.

In this study, we reveal the ideal electrochemical potential factor and reaction mechanism of RMs for the efficient direct recycling of spent cathodes in LIBs. We chose 3,5‐di‐tert‐butyl‐o‐benzoquinone (DTBQ) as the RM to understand the RM‐assisted relithiation process and develop a straightforward immersion technique for non‐homogenous and Li‐deficient cathode electrodes (LiNi_0.6_Co_0.2_Mn_0.2_O_2_, NCM622). As detailed in **Figure**  **1**, the Li replenishment to Li‐deficient cathodes occurs via redox mediation between the spent cathodes and the reductive DTBQ‐based solution. We elucidated the structural changes in spent cathode materials during RM‐assisted chemical relithiation. We further investigated the structural and chemical changes in DTBQ during the chemical relithiation reaction using in situ spectroscopy and density functional theory (DFT) calculations. Our study provides insights into the development of potential RM candidates to uniformize the Li distribution in used cathodes for realizing the effective direct recycling of spent LIBs.

## 2 Results and Discussion

**Figure 2:**
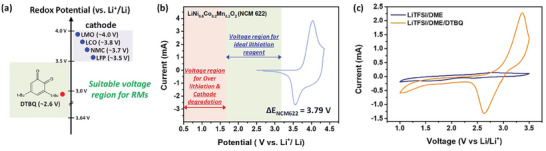
(a) Illustration of the redox potentials of cathode materials and DTBQ, (b) CV profiles of NCM622 and the illustration of the voltage region for the ideal lithiation reagent, and (c) CV profiles of LiTFSI/DME, and LiTFSI/DME/DTBQ solutions.

In the RM‐assisted chemical lithiation process, DTBQ acts as a redox mediating agent, transferring Li ions and electrons to the Li‐deficient cathode materials. When the Li metal is immersed in a DTBQ‐containing solution, DTBQ binds to the Li ions dissolved from the Li metal and is subsequently reduced (Figure  1). When Li‐deficient cathode materials are immersed in a Li‐coordinated DTBQ solution, the Li‐coordinated DTBQ molecules are oxidized, transferring Li ions and reducing themselves. Li‐ion transfer from RMs to cathodes occurs when the electrochemical potential (which has an inverse relationship with the electron energy) of RMs is lower than that of the cathode materials, indicating the importance of the electrochemical potential level of RM molecules. Another considerable factor is that the redox potential of RMs should be higher than ≈2 V because the extremely low potential of RMs tends to further reduce and decompose the cathode materials owing to overlithiation (e.g., LiCoO_2_ + Li^+^ + e^−^ → Li_2_O + Co + 1/2O_2_). To evaluate the suitability of DTBQ as an effective RM for the regeneration of Li‐deficient cathodes, we performed a comparative analysis of the electrochemical redox potential of DTBQ against those of various cathode materials, including lithium manganese oxide, lithium cobalt oxide, lithium nickel manganese cobalt oxide (NMC), and lithium iron phosphate (**Figure**  **2**a).^[^ ^15^ ^]^ The DTBQ RMs exhibited lower redox potentials than the transition metal redox potentials of conventional cathode materials, confirming that the DTBQ molecule is a suitable candidate as an RM additive.

Cyclic voltammetry (CV) tests were conducted on the NCM622 cathodes (P‐NCM) to identify a suitable electrochemical potential range for the lithiation RM reagent (Figure  2b). The redox potential of NCM622 is 3.79 V. According to previous studies, the NCM622 cathodes undergo decomposition and conversion reactions when subjected to voltages below 1.64 V (vs Li^+^/Li). Consequently, the redox potential for an ideal lithiation RM reagent should range between 1.64 and 3.3 V. CV tests were performed to evaluate the redox potential of DTBQ (Figure  2c). DTBQ exhibited a redox potential of 2.6 V, which is appropriate for the effective lithiation of Li‐deficient NCM622 cathodes. Finally, we selected DTBQ as the reductive lithiation agent to regenerate the spent NCM622 cathode materials.

**Figure 3:**
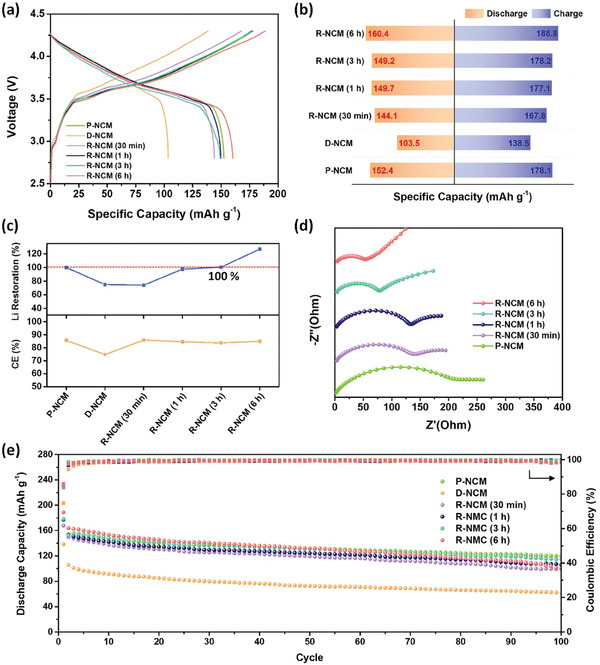
(a) Initial charge–discharge curves of the P‐NCM, D‐NCM, R‐NCM (30 min), R‐NCM (1 h), R‐NCM (3 h), and R‐NCM (6 h), (b) Initial charge–discharge capacities of the P‐NCM, D‐NCM, R‐NCM (30 min), R‐NCM (1 h), R‐NCM (3 h), and R‐NCM (6 h), (c) Li restoration and Coulombic efficiency of the P‐NCM, D‐NCM, R‐NCM (30 min), R‐NCM (1 h), R‐NCM (3 h), and R‐NCM (6 h), (d) EIS spectra of the P‐NCM, D‐NCM, R‐NCM (30 min), R‐NCM (1 h), R‐NCM (3 h), and R‐NCM (6 h) after DTBQ‐assisted chemical relithiation, (e) Cycling performance of the P‐NCM, D‐NCM, R‐NCM (30 min), R‐NCM (1 h), R‐NCM (3 h), and R‐NCM (6 h).

We adopted the direct immersion method for a Li‐deficient NCM622 (D‐NCM) electrode into a lithiated DTBQ solution instead of immersing the cathode powder. This approach enables the direct recycling of the cathodic electrode without eliminating the binder and conductive carbon agent from the electrode. The electrochemical performances of P‐NCM, D‐NMC, and R‐NCM (30 min, 1 h, 3 h, and 6 h) collected at each processing step were examined to confirm the effect of the DTBQ‐assisted chemical relithiation process (**Figure**  **3**). Figure  3a shows the initial charge–discharge curves of the NCM electrode samples collected under different treatment conditions. This assessment was conducted using full‐cell tests to investigate the influence of reaction time on the degree of chemical relithiation.

After regeneration, capacity recovery was observed without charge/discharge curve deformation, indicating the successful lithiation of the cathode materials without notable side reactions. The P‐NCM exhibited the initial charge and discharge capacities of 178.1 and 152.4 mAh g^−1^, respectively. We intended to extract Li ions from the P‐NCM to prepare the D‐NMC with a charge capacity of ≈75% (Figure S1, Supporting Information). As the reaction time for relithiation increased, the charge capacity gradually recovered, approaching that of the P‐NCM. For the R‐NCM (3 h), the initial charge capacity was 178.2 mAh g^−1^, leading to the full capacity of the P‐NCM as the optimized condition (Figure  3b,c). However, overlithiation occurred in the case of relithiation for 6 h because of additional surface‐side reactions between the Li sources and electrolyte species, such as the formation of a cathode–electrolyte interface layer. Furthermore, batteries that have been disassembled or discarded after prolonged use often contain cathode materials exhibiting substantial capacity loss, structural damage, and cation disorder. Therefore, the proposed DTBQ‐assisted chemical relithiation was evaluated to confirm its effectiveness in restoring the electrochemical performance and structural recovery of highly degraded NCM (HD‐NCM). HD‐NCM shows a significant reduction in both specific capacity and voltage compared to the P‐NCM, indicating the severity of degradation caused by Li deficiency and structural changes (Figure S2, Supporting Information). After chemical relithiation for 3 h, the R‐NCM exhibits a substantial recovery in both capacity and voltage, closely approaching the performance of P‐NCM.

To further understand the enhanced cycling stability of R‐NCM, the dQ/dV curves of HC‐NCM were compared with those of the P‐NCM and R‐NCM (Figure S3, Supporting Information). For NCM622, the initial hexagonal (H1) phase transitions to a monoclinic (M) phase and subsequently to a secondary hexagonal (H2) phase during the extraction of Li+ ions. The transformation from the H1 phase to the M phase corresponds to the oxidation of Ni^2+^/Ni^3+^ to Ni^4+^, with the oxidation peak occurring ≈3.75 V.^[^ ^16^ ^]^ HD‐NCM shows higher oxidation voltages and lower reduction voltages compared to P‐NCM, with increased gaps between the charge and discharge plateaus. These changes are caused by the structural transformation from the layered phase to the rock‐salt phase during the charge/discharge process. This evolution is also reflected in the dQ/dV curve as peak broadening, voltage shifts, indicating increased resistance and polarization. Interestingly, the dQ/dV curve of the relithiated R‐NCM is reversibly retrieved to that of the P‐NCM, indicating a successful recovery of the layered structure after the chemical relithiation process. It suggests that the key electrochemical features, including the redox peaks associated with the sequential oxidation and reduction have been restored.

To investigate the interfacial stability and kinetic differences in Li^+^ diffusion for the P‐NCM, D‐NCM, and R‐NCM, electrochemical impedance spectroscopy (EIS) measurements were conducted (Figure  3d). The high‐frequency semicircle is mainly related to the charge‐transfer resistance between the electrolyte and the electrode.^[^ ^17^ ^]^ The straight line at low frequencies represents the Warburg impedance associated with the diffusion of Li ions.^[^ ^18^ ^]^ Before chemical relithiation, the charge‐transfer resistance (R_ct_) value of the P‐NCM was 212 Ω. High‐Ni cathodes exhibit high reactivity and instability when exposed to air and ambient humidity. This exposure leads to the reactions of H_2_O and CO_2_ with Li^+^ ions, forming lithium carbonate and hydroxide species. Consequently, this surface structural degradation increases the impedance.^[^ ^19^ ^]^ After chemical relithiation, a significant decrease in R_ct_ was observed with increasing reaction time. Therefore, the R‐NCM (6 h) showed the lowest R_ct_ value of 53.6 Ω. The reduced R_ct_ value indicates that lithiation for 6 h can effectively facilitate charge transfer at the surface owing to the structural and compositional recovery of the cathode materials. The cycle performance of various NMC cathode electrodes before and after relithiation was evaluated at 0.1 C in a full‐cell configuration (Figure  3e). Among them, the R‐NCM (3 h) exhibited excellent capacity retention (76.7%), comparable to that of the P‐NCM (78.3%) after 100 cycles, whereas the other R‐NCM samples showed a relative deterioration of the capacity value for cycling. These results demonstrate that chemical relithiation was successfully achieved without performance degradation. Therefore, the DTBQ‐assisted chemical relithiation approach is an effective method to directly regenerate NCM cathodes.

**Figure 4:**
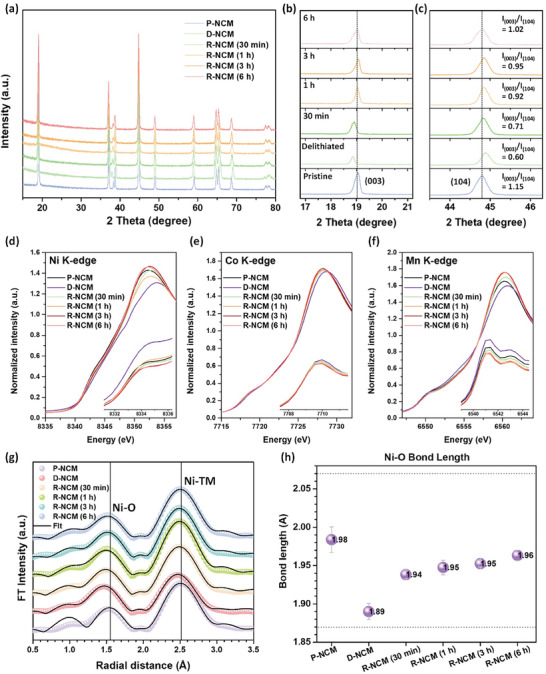
(a) Ex situ XRD patterns of the P‐NCM, D‐NCM, R‐NCM (30 min), R‐NCM (1 h), R‐NCM (3 h), and R‐NCM (6 h) (b) Enlargement of the regions in the range of 16.8°–21.2° (c) Enlargement of the regions in the range of 43.5°– 46.3° (d) Normalized TM K‐edge XANES spectra of Ni K‐edge (e) Co K‐edge, and (f) Mn K‐edge. (g) Fourier‐transformed magnitudes of *k^2^* weighted Ni K‐edge EXAFS spectra with the fitting results. (h) Changes in the Ni‐O bond length obtained by the curve fitting analysis of the Ni K‐edge EXAFS spectra.

To elucidate the structural recovery of the NCM cathode after the DTBQ‐assisted chemical relithiation, X‐ray diffraction (XRD) was performed for different relithiation states. All the samples showed a hexagonal R‐3 m space group of the α‐NaFeO_2_ structure (**Figure**  **4**a).^[^ ^20^ ^]^ In the D‐NCM, the (003) peak shifted to lower angles, whereas the (104) peak shifted to higher angles compared with those of the P‐NCM, indicating the expansion and contraction of the *c*‐ and *a*‐lattice parameters, respectively. These changes were attributed to the increased electrostatic repulsion between the oxygen layers and the reduced ionic radius associated with the partial oxidation of Ni^2+^ to Ni^3+^ in the Li‐deficient state (Figure  4b,c).^[^ ^21^ ^]^ The intensity ratio of (003)/(104) indicates the degree of cation mixing between Li and the transition metals.^[^ ^22^ ^]^ The D‐NCM exhibited the lowest (003)/(104) intensity ratio, corresponding to a higher degree of cation mixing (Figure  4c). Furthermore, the degree of peak splitting for the (006)/(102) and (108)/(110) increased after delithiation (D‐NCM) and subsequently recovered during lithiation, indicating a reversible crystalline restoration of the hexagonal layered structure (Figure S4, Supporting Information). Following the regeneration process, all the peaks gradually returned to their original positions, indicating the recovery of their crystal structures. Specifically, the (003) peak shifted toward a higher angle and the (104) peak shifted toward a lower angle, indicating the recovery of ordered layered structures. These peak shifts indicate the refilling of Li vacancies, leading to a decrease in the *c*‐axis, whereas the *a*‐lattice parameter increased owing to the reduction of high‐valence Ni to a lower oxidation state, which has a larger ionic radius. Additionally, the increased degree of peak splitting of the (006)/(102) and (108)/(110) peak pairs and the higher peak ratio of (003)/(104) indicate a decrease in Li‐deficient sites upon Li supplementation, which is consistent with the R‐NCM samples.^[^ ^23^ ^]^ The reduced degree of cation mixing indicates a highly ordered layered structure in the R‐NCM samples. To further demonstrate the structural recovery of HD‐NCM, the DTBQ‐assisted chemical relithiation process was applied to HD‐NCM samples, which exhibited severe structural distortion and cation disorder due to long‐term cycling and significant Li deficiency. After chemical relithiation, the XRD peaks of HD‐NCM returned to their original positions, indicating the restoration of the crystalline structure (Figures S5 and S6, Supporting Information). It confirms that the DTBQ‐assisted chemical relithiation process effectively restores the reversible structure restoration of highly degraded NCM, validating its applicability for cathode materials with highly degradation.

To further investigate the surface chemical structures after chemical relithiation, ex situ X‐ray photoelectron spectroscopy (XPS) was performed before and after regeneration (Figure S7, Supporting Information). The Ni 2p_3/2_ peak was deconvoluted into Ni^2+^ (855.03 eV) and Ni^3+^ (856 eV) peaks, and satellite peaks were observed at 860.5 eV owing to shake‐up processes (Figure S7a, Supporting Information). In the D‐NCM, the intensity of Ni^2+^ (64.8%) significantly increased compared with that in the P‐NCM (31.6%). This is anticipated because Ni^4+^, formed during the delithiation process, receives electrons from lattice oxygen and is reduced to Ni^2+^, which can result in the formation of NiO on the surface (Figure S7b, Supporting Information).^[^ ^24^ ^]^ However, in the R‐NCM (3 h) sample, the content of Ni^2+^ was significantly reduced to 34.4%, similar to the P‐NCM results. The conversion of Ni^2+^ to Ni^3+^ strongly supported the restoration of the impurity phase to a highly ordered layered structure, indicating the effective repair of the surface and bulk structures during the chemical relithiation process.^[^ ^21^ ^]^

It is crucial to reveal the oxidation state of the transition metal and the local structural changes to support reversible relithiation assisted by DTBQ RMs. Ex situ X‐ray absorption spectroscopy spectra at the Ni, Co, and Mn K‐edges of the P‐NCM, D‐NCM, and R‐NCM electrodes were measured as bulk structural probes (Figure  4d–f). The K‐edge spectrum is divided into two regions: i) X‐ray absorption near edge structure (XANES) and ii) extended X‐ray absorption fine structure (EXAFS) regions. The XANES spectra prove the coordination environment and oxidation state of the element, whereas the EXAFS spectra provide local structural information, including bond lengths, coordination numbers, and atomic disorder (i.e., Debye–Waller factor), in an elemental‐selective manner.^[^ ^25^ ^]^ The normalized Ni, Co, and Mn K‐edge XANES spectra are shown in Figure  4d–f, and the magnified pre‐edge (1s→3d transitions) region is shown in the insets.^[^ ^26^ ^]^ In the Ni K‐edge spectra, the D‐NCM exhibited an absorption edge (1s→4p transitions) energy shift toward higher energy, indicating an increased oxidation state of Ni in the D‐NCM.^[^ ^27^ ^]^ Furthermore, the pre‐edge peak in the D‐NCM, associated with a dipole‐forbidden transition, significantly increased because of the increased local structural distortion in the NiO_6_ octahedra and the corresponding increased p‐d orbital mixing character.^[^ ^28^ ^]^ When Li is replenished via chemical relithiation, the spectral features and oxidation state of Ni ions are recovered to those of the P‐NCM, indicating the structural restoration of the D‐NCM by Li supplementation. Peak changes were observed in the Mn K‐edge spectra, similar to those observed for Ni. The intensity of the main edge peak decreased, whereas the pre‐edge intensity increased, implying that the local structural distortion around Mn intensified in the Li‐deficient environment in the D‐NCM. After Li regeneration, the main‐edge and pre‐edge intensities were retrieved, verifying the mitigated lattice distortion. In contrast, the position of the Co K‐edge showed a negligible peak shift, indicating that Co^3+^ remained unchanged during the Li extraction and restoration processes.

The ex‐situ EXAFS spectra were analyzed to investigate the local chemical and structural changes in Ni, Co, and Mn, and the transition metal–oxygen (TM–O) and TM–TM interatomic distances. The Fourier‐transformed (FT)‐EXAFS spectra (not phase‐corrected, causing shorter bond lengths in the plots than for the real ones by ≈0.3–0.4 Å)^[^ ^28^ ^]^ are shown in Figures  4g and S8 (Supporting Information). The first peak ≈1.5 Å corresponds to the TM–O interaction, whereas the second peak ≈2.5 Å is related to the TM–TM interactions.^[^ ^26^ ^]^ The local structure around Co for all the samples showed no noticeable variations (Figure S8, Supporting Information), consistent with the Co XANES results. In the Mn K‐edge EXAFS spectra, the Mn‐O peak intensity decreased in the D‐NMC without a discernible peak shift, revealing an increased local structural distortion while maintaining the Mn^4+^ states in a Li‐deficient environment. After Li regeneration, the Mn‐O peak intensities were retrieved, verifying the recovered local distortion in the R‐NCM, which coincided with the Mn XANES results. However, notable peak shifts and intensity variations were observed in the Ni EXAFS spectra (Figure  4g). Therefore, a curve fitting analysis was performed to obtain quantitative structural information, including the Ni‐O bond length, using a model layered LiNiO_2_ structure, as shown in Figure  4h. The fitted structural parameters are presented in Table S1 (Supporting Information). The Ni‐O bond length in the P‐NCM was ≈1.98 Å. In contrast, the D‐NCM exhibited a much shorter bond length of ≈1.89 Å, confirming a substantial presence of higher Ni oxidation states (i.e., Ni^4+^) after delithiation. Moreover, upon supplementation with Li (R‐NCM), the average Ni oxidation state decreased reversibly, resulting in a bond length similar to that observed for the P‐NCM (Figure  4h). These results indicate reversible changes in the oxidation states and structural configurations of the transition metals in the spent NCM cathodes during the regeneration process.

**Figure 5:**
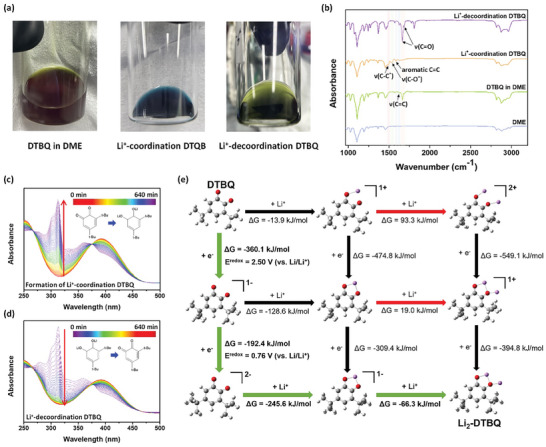
(a) Photographs of the DTBQ in DME, Li^+^‐coordination DTBQ (contact Li metal with DTBQ in DME), and Li^+^‐decoordination DTBQ (after relithiation reaction) (b) FT‐IR spectra of DME, DTBQ in DME, Li^+^‐coordination DTBQ, and Li^+^‐decoordination DTBQ solutions (c) UV–vis spectra of (c) Li^+^‐coordination DTBQ and (d) Li^+^‐decoordination DTBQ solutions at different times. (e) Calculated reaction Gibbs free energy and favored pathway (green arrows). The redox potential for the reduction step within this favored route has also been computed. Notably, ΔC_2Li_ equals 1, signifying the augmentation of Clar's sextet following dual lithiation events. Color index: Grey – C, white – H, oxygen – H, purple – Li.

Investigating the chemical and structural changes in DTBQ RMs by Li^+^ coordination and decoordination is important for understanding the DTBQ‐assisted relithiation mechanisms and for the structural investigation of NMC cathodes. We performed Fourier‐transform infrared (FT‐IR) spectroscopy, UV–vis spectroscopy, and molecular orbital energy calculations, as shown in **Figure**  **5**a–d. The optical appearance and color change in the DTBQ‐containing solution are direct indicators of Li^+^ coordination and decoordination (Figure  5a). The DTBQ in the 1,2‐dimethoxyethane (DME) solution was dark yellow. After the immersion of Li metal in the DTBQ solution, the color of the solution changed to blue, indicating the Li^+^‐coordination of the DTBQ solution. After the relithiation reaction, the solution became dark green, corresponding to the Li^+^‐decoordinated DTBQ solution.

Figure  5b shows the FT‐IR spectra of the DTBQ‐containing DME solutions under different Li^+^ coordination and decoordination conditions. The FT‐IR spectra of DTBQ in DME show the stretching vibrations of v(C = O) at 1686 and 1665 cm^−1^ and the absorption peaks of v(C = C) at 1624 cm^−1^ originating from DTBQ.^[^ ^13a^ ^]^ For Li^+^‐coordinated DTBQ, the typical C = O and C = C peaks of DTBQ disappeared upon Li coordination. Concurrently, new peaks corresponding to C‐O^*^ and C‐C^*^ radicals and aromatic C = C peaks were observed. The appearance of radical peaks resulted from the coordination of the oxygen atoms in DTBQ with Li ions, whereas the aromatic C = C peak emerged owing to the electrochemical reduction of quinone molecules. These results demonstrate that DTBQ is chemically reduced by Li^+^ ions and electron pairs at the Li surface. After the spent cathode was immersed in a Li^+^‐coordinated DTBQ solution, the aromatic C = C and C‐O^*^ peaks disappeared. In addition, the C = O and C = C peaks corresponding to pristine DTBQ were observed. A weak C‐C^*^ peak remained, which is attributed to the small amount of Li^+^‐coordinated DTBQ molecule residues remaining in the Li^+^‐decoordinated DTBQ solution, even after chemical relithiation.

To further investigate the DTBQ‐assisted relithiation mechanism, we conducted in situ UV–vis spectrophotometry analysis and monitored the structural changes in DTBQ depending on the coordination of Li^+^ ions collected at different times. After immersing the Li metal in a DTBQ‐containing DME solution, the progress of the reaction was monitored by recording the UV–vis spectra of the solution mixture (Figure  5c). A UV–vis absorption peak was observed at 407 nm, corresponding to DTBQ.^[^ ^29^ ^]^ The peak gradually decreased and new peaks appeared between 300 and 325 nm after exposure to the Li metal. This was primarily due to the consumption of DTBQ and the increased concentration of Li^+^‐coordinated DTBQ. After immersing the Li‐deficient cathode in the mixture, the peak at 300–325 nm gradually decreased and a DTBQ peak was retrieved, indicating the reversible conversion of Li^+^‐coordinated DTBQ to DTBQ during the chemical relithiation reaction (Figure  5d). These results were further confirmed using ex situ UV–vis analysis (Figure S9, Supporting Information). After repeatedly immersing several Li‐deficient cathodes, the color of the solution returned to its original dark yellow state. These results demonstrate that the Li metal can be immersed in a used solution, which can then be reused as a regeneration solution. The structural and chemical characterizations of the Li^+^‐coordinated reagents, depending on the degree of lithiation, confirmed that the chemical relithiation of the spent cathode via the assistance of DTBQ occurs reversibly.

DFT calculations were performed to elucidate the reaction mechanism underlying DTBQ's role as an RM. Electronic structure analyses were conducted across all conceivable structures using the IEFPCM implicit solvation model framework.^[^ ^30^ ^]^ Specifically, The lithiation process was dissected into two distinct phases: reduction and interaction with Li^+^. The calculations targeted several potential intermediate complexes, including DTBQ^−^, DTBQ^2−^, Li‐DTBQ^+^, Li‐DTBQ, Li‐DTBQ^−^, Li_2_‐DTBQ^2+^, and Li_2_‐DTBQ^+^, which can be considered for the reaction DTBQ + 2Li^+^ + 2e^−^ ⇌ Li_2_‐DTBQ. The Gibbs free energy of each complex was calculated by incorporating zero‐point energy and thermal corrections. Thermodynamically more stable reactions were identified based on the more negative ΔG value. These computational analyses revealed a sequential reaction pathway, which involves two reduction steps followed by two complex formation processes through interactions with Li^+^ ions, as depicted in Figure  5e (i.e., DTBQ → DTBQ^−^ → DTBQ^2−^ → Li‐DTBQ^−^ → Li_2_‐DTBQ).

Meanwhile, Pekka and Hubert have critiqued the reliance on the highest occupied molecular orbital and lowest unoccupied molecular orbital energies for predicting redox reactions as imprecise.^[^ ^31^ ^]^ Instead, they recommended investigating the redox potential using the Gibbs free energy differential between the reactants and products. Similar to recent studies that successfully predicted the redox potential in a solution using the Born–Haber cycle approach based on the Gibbs free energy,^[^ ^32^ ^]^ we applied the same methodology to predict the redox potential of the redox reactions. The predicted redox potential for the DTBQ + e^−^ ⇌ DTBQ reaction is 2.50 V versus Li/Li^+^, and for the DTBQ^−^ + e^−^ ⇌ DTBQ^2−^ reaction is 0.76 V versus Li/Li^+^ (as shown in Figure  5e). These values are positioned between the redox potentials of the lithium metal anode and the NCM622 cathode.^[^ ^33^ ^]^ Notably, the predicted redox potential of the first reduction reaction was closely aligned with the peak position observed in the electrochemical analysis, as shown in Figure  2c.

These experimental findings align with those of a prior study conducted by Dihua et al., who utilized Clar's rule to predict the lithiation voltages of carbonyl‐containing polycyclic aromatic hydrocarbon materials.^[^ ^34^ ^]^ An examination of the calculated structures for DTBQ and Li_2_‐DTBQ depicted in Figure  5e shows an increase in the number of Clar's sextets from 0 to 1, corresponding to ΔC_2Li_ = 1 in their terminology. Under these conditions, the anticipated redox voltage versus Li/Li^+^ was estimated to be between 2.5 and 2.75 V, corroborating the experimental observations. This phenomenon can be attributed to stabilization through the delocalization of electrons. Our study demonstrates a novel and efficient approach for the direct recycling of spent NCM cathodes using DTBQ as an RM, achieving uniform Li replenishment, structural restoration, and enhanced electrochemical performance, thereby offering a sustainable, environmentally friendly, and cost‐effective alternative to traditional recycling methods for Li‐ion batteries.

## 3 Conclusion

In summary, we elucidated the potential energy factor and reaction mechanism of DTBQ RMs for RM‐assisted chemical relithiation to regenerate homogeneous Li and the structure of spent NCM622 cathodes as an effective direct recycling strategy. By examining the electrochemical potential, we confirmed that DTBQ is an appropriate RM, which can effectively supplement the insufficient Li in the spent cathode material. The direct immersion technique of non‐homogenous and Li‐deficient NCM cathodes was used for a straightforward and facile chemical relithiation process. We achieved 100% relithiation efficiency and showed excellent cycle performance by conducting DTBQ‐assisted chemical relithiation for 3 h. From advanced structural characterization, we confirmed a reversibly recovered crystalline structure and chemical status of the NCM622 after DTBQ‐assisted chemical relithiation. Finally, we successfully demonstrated the detailed reaction mechanism of DTBQ redox molecules (DTBQ + 2Li^+^ + 2e^−^ ⇌ Li_2_‐DTBQ) accompanied by their structural and chemical reversibility using in situ spectroscopy and DFT calculations. Our study allows a better understanding of seeking suitable RM candidates for chemical relithiation as a sustainable and efficient direct recycling approach for spent LIB cathodes.

## 4 Experimental Section

### Relithiation Process of Cathode Material

Chemically delithiated NCM622 (LiNi_0.6_Co_0.2_Mn_0.2_O_2_) with ≈25% Li loss was used as the spent cathode (D‐NCM) material. After two pre‐cycles at 0.5 C (1 C = 180 mA g^−1^) in the full cell with a graphite anode (n/p ratio = 1.1), the D‐NCM sample was charged to extract ≈25% of the lithium relative to its capacity from the last discharge. Cells were detached with a nipper, and the electrode was retrieved using tweezers. The collected electrodes were washed with ethylene carbonate (EC). DTBQ in the DME electrolyte (0.5 m) was prepared and stirred overnight. Subsequently, Li metal was added to the electrolyte to obtain a lithiated DTBQ solution. Before immersing the collected electrodes, the Li metal was removed from the solution. The collected electrodes were then immersed in the lithiated DTBQ solution for different durations (30 min, 1 h, 3 h, and 6 h). The sampling was conducted in an Ar‐filled glovebox.

### Material Characterization

The crystalline phases of the sample were characterized using XRD (D8 Advance, Bruker) with Cu‐Kα (λ = 1.54 Å) radiation. The surface atomic chemical compositions were analyzed using XPS (K‐alpha, Thermo U.K.). A UV–vis spectrophotometer (UV‐2550, Shimazu) and an FT‐IR spectrophotometer (Nicolet IS50, Thermo Fisher Scientific) were used to investigate the DTBQ‐assisted relithiation mechanism. The structural changes and surface characteristics of the electrodes were assessed using *ex‐situ* XRD and XPS. The electrodes were prepared using DTBQ‐assisted chemical relithiation.

### Electrochemical Characterization

The cells were assembled in an Ar‐purged glove box (O_2_ and H_2_O < 0.1 ppm, MBraun, Korea). The full cell consisted of a graphite anode and an NCM622 cathode prepared with an N/P ratio of 1.1. The electrolyte was 1 M LiPF_6_ in EC and diethyl carbonate (v/v = 1:1), and the volume in each cell was maintained at 90 µL. Celgard 2500 polypropylene (19 mm diameter) was used as the separator. The electrochemical performance measurements were conducted using a potentio‐galvanostat (WBCS3000, WonATech, Korea) at a constant current density of 18 mA g^−1^ (0.1 C) within the voltage range of 2.8–4.3 V versus Li/Li^+^. All the cell tests were conducted after two formation cycles at 0.1 C within the voltage range of 2.8–4.3 V. The highly degraded NCM was first cycled for 50 cycles at 0.5 C, followed by further delithiation with 20% Li extraction. A Biologic VSP potentiostat was used for the CV experiments at a scan rate of 30 mV s^−1^ and EIS measurements. EIS was measured between 100 kHz and 0.01 Hz with an amplitude of 5 mV.

### X‐Ray Absorption Spectroscopy

Ni, Co, and Mn K‐edge XAS spectra were collected at the 7D and 8C beamlines at the Pohang Light Source (PLS‐II) using a Si(111) monochromator in transmission mode at room temperature. The cathode electrodes for the X‐ray absorption spectroscopy (XAS) measurements were sealed with Kapton tape after washing and drying with the DMC solvent under an Ar flow. The XAS data were processed using the Athena and Artemis programs. To obtain the magnitude plots of the EXAFS spectra in an R‐space (Å), the k^2−^weight signal was used and Fourier‐transformed with a *k*‐range of 3.1–11.0 Å^−1^ using a Hanning window function. The curve fitting analysis was performed in the R‐range of 1.0–3.0 Å, covering the first Ni‐O shells and second Ni‐TM shells, using a single scattering algorithm generated with the FEFF 6.0 ab‐initio simulation code based on the LiNiO_2_ structure. The amplitude factor (S_0_2) was fixed at 0.7 (0.6 for the D‐NCM). The EXAFS R‐factors were less than 0.2% in all cases.

### DFT Calculations

All the quantum calculations were performed using the Gaussian 16 computational suite.^[^ ^35^ ^]^ Structure optimizations and energy calculations were performed using DFT with the M06‐2X exchange‐correlation functional and the def2‐TZVP basis set.^[^ ^36^ ^]^ This functional‐basis set combination has been shown to provide an accuracy comparable to that of CCSD(T) calculations, which were widely regarded as the gold standard in quantum chemistry.^[^ ^37^ ^]^ The implicit solvation effects were modeled using the IEFPCM solvation model, with a dielectric constant (ε) of 7.2 to simulate DME as the solvent.^[^ ^30^, ^38^ ^]^

The redox potential of the species in the solution environment was calculated based on the following equation using the Nernst equation and Hess's law:^[^ ^32^, ^39^ ^]^

(1) $$E_{red}\left(\mathrm{versus}\;\mathrm{Li}\;/\;Li^{+}\right)=-\frac{\Delta E_{a}+\Delta G_{s}\left(A^{-}\right)-\Delta G_{s}\left(A\right)}{F}-1.23$$

Here, ΔE_a_ is the electron attachment energy (electron affinity) at 0 K, ΔG_s_(*A* ^−^) is the solvation‐free energy of the reduced structure, ΔG_s_(*A*) is the solvation‐free energies of the initial structures, and F is the Faraday constant (96 485 C mol^−1^). A shift factor of 1.23 was used to correct for the difference between the absolute potential scale and Li/Li^+^.^[^ ^32b^ ^]^ The redox potential of the species was compared with the Fermi level or open‐circuit voltage of the electrode, and the redox reaction owing to electron transfer from the electrode to the species or from the species to the electrode was predicted by considering the chemical potential of the electrons.

## Conflict of Interest

The authors declare no conflict of interest.

## Supporting information



Supporting Information

## Data Availability

The data that support the findings of this study are available from the corresponding author upon reasonable request.
