# Supplementary material for: Unraveling Redox Mediator‐Assisted Chemical Relithiation Mechanism for Direct Recycling of Spent Ni‐Rich Layered Cathode Materials
Source: Adv Sci (Weinh). 2025 Jan 22;12(11):2417094. doi: 10.1002/advs.202417094 (PMC11923935; doi:10.1002/advs.202417094)
Supplement: Supplementary file 1 — Supporting Information [file ADVS-12-2417094-s001.docx]

Supporting Information

Unraveling Redox Mediator-Assisted Chemical Relithiation Mechanism for Direct Recycling of Spent Ni-rich Layered Cathode Materials

*Suji Kim^1,†^, Ukseon Shin^2,†^, Hyun Ju Yoon^1^, Soo-Ah Yoon^3^, Jinju Song^4^, Jiyoung Ma^4^, Jung-Je Woo^4^, Kyung-Wan Nam^3,*^, Dong-Hwa Seo^2,*^, and Won-Hee Ryu^1,*^*

Suji Kim, Hyun Ju Yoon, Won-Hee Ryu

^1^Department of Chemical and Biological Engineering,
Sookmyung Women’s University,
100 Cheongpa-ro 47-gil, Yongsan-gu, Seoul 04310, Republic of Korea
E-mail: whryu@sookmyung.ac.kr (Corresponding author)

Ukseon Shin, Dong-Hwa Seo
^2^Department of Materials Science and Engineering,
Korea Advanced Institute of Science and Technology (KAIST),
291 Daehak-ro, Daejeon 34141, Republic of Korea
E-mail: dseo@kaist.ac.kr (Corresponding author)

Soo-Ah Yoon, Kyung-Wan Nam
^3^Department of Energy & Materials Engineering
Dongguk University
Seoul 04620, Republic of Korea
E-mail: knam@dongguk.edu (Corresponding author)

Jinju Song, Jiyoung Ma, Jung-Je Woo
^4^Gwangju Clean Energy Research Center
Korea Institute of Energy Research (KIER)
270-25 Samso-ro, Gwangju 61003, Republic of Korea

^†^ These authors contributed equally to this work.


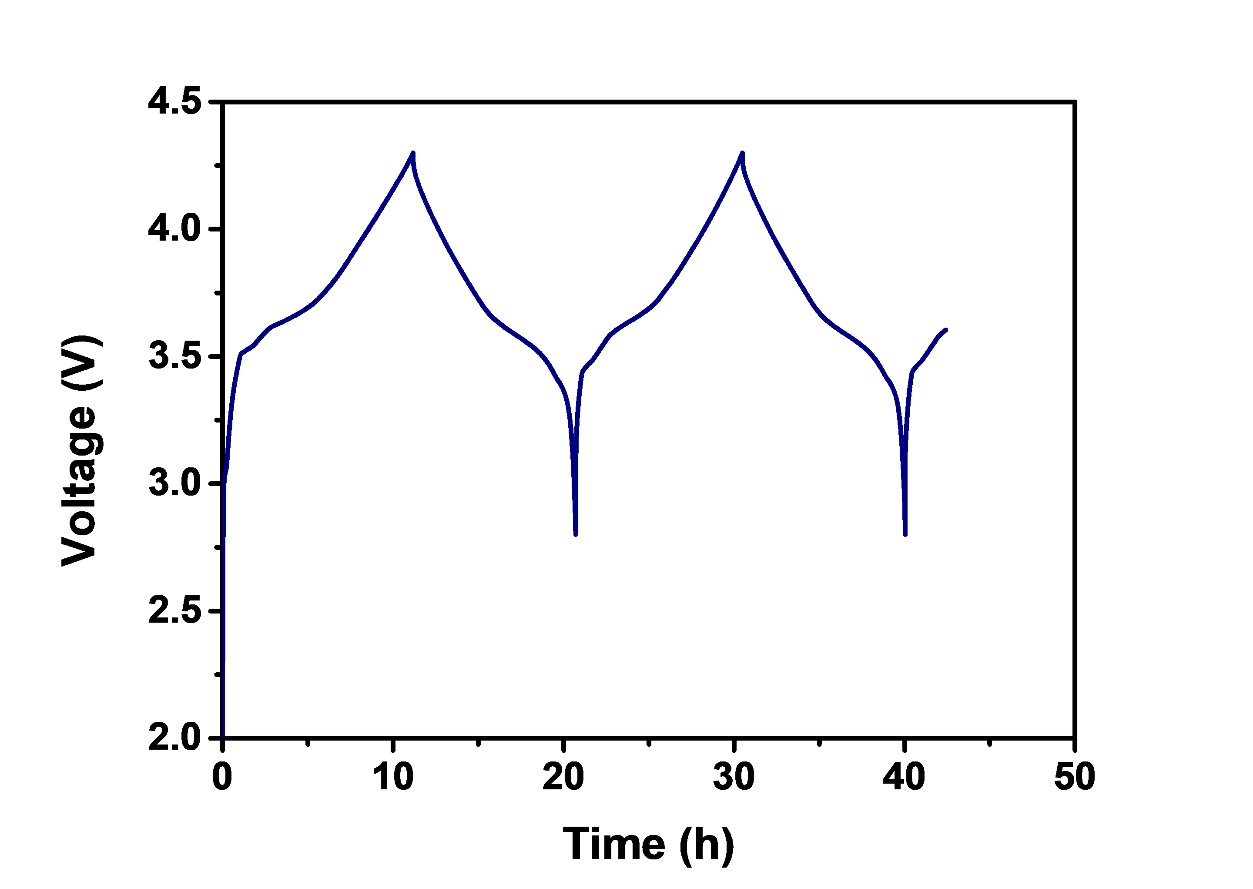


**Figure S1.** Voltage–time curves for the prepared D-NCM sample (approximately 25% Li extraction)


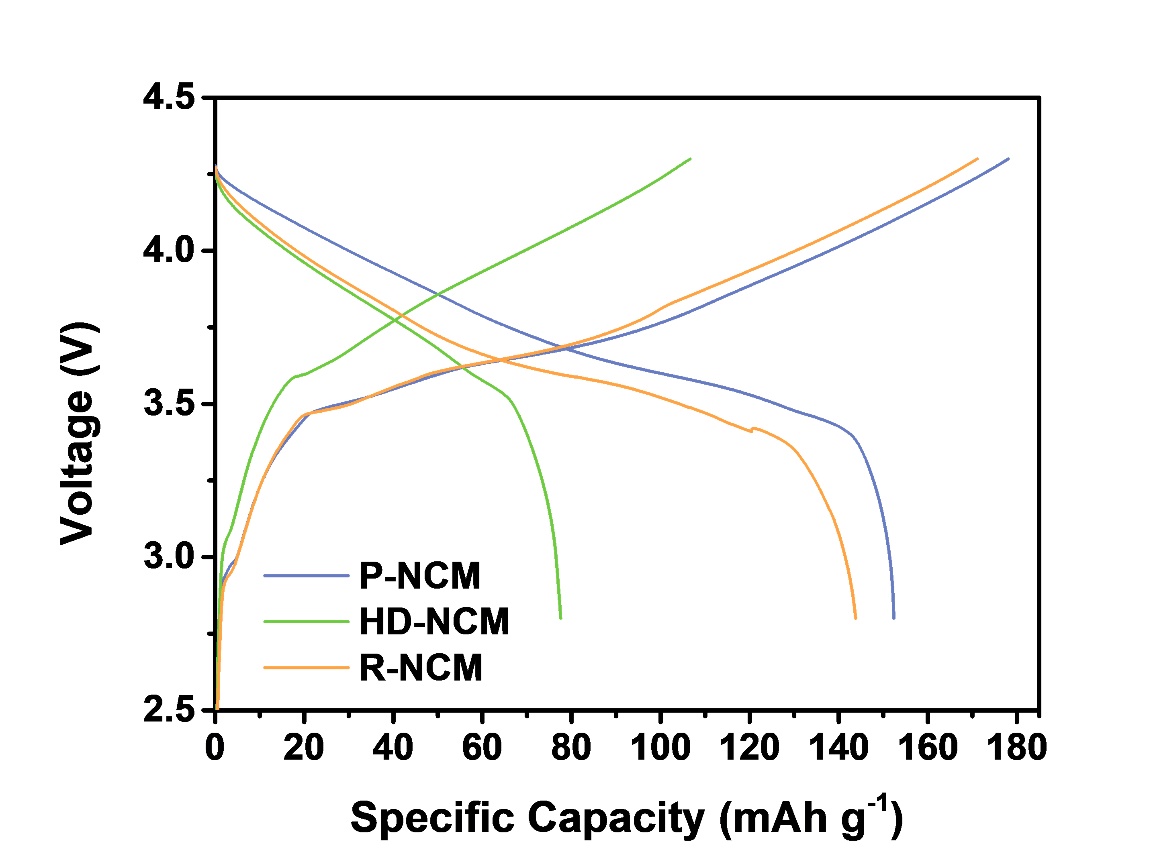


**Figure S2.** Initial charge–discharge curves of the P-NCM, HD-NCM, and R-NCM

**Figure S3.** dQ/dV vs. Voltage curves for P-NCM, HD-NCM, and R-NCM


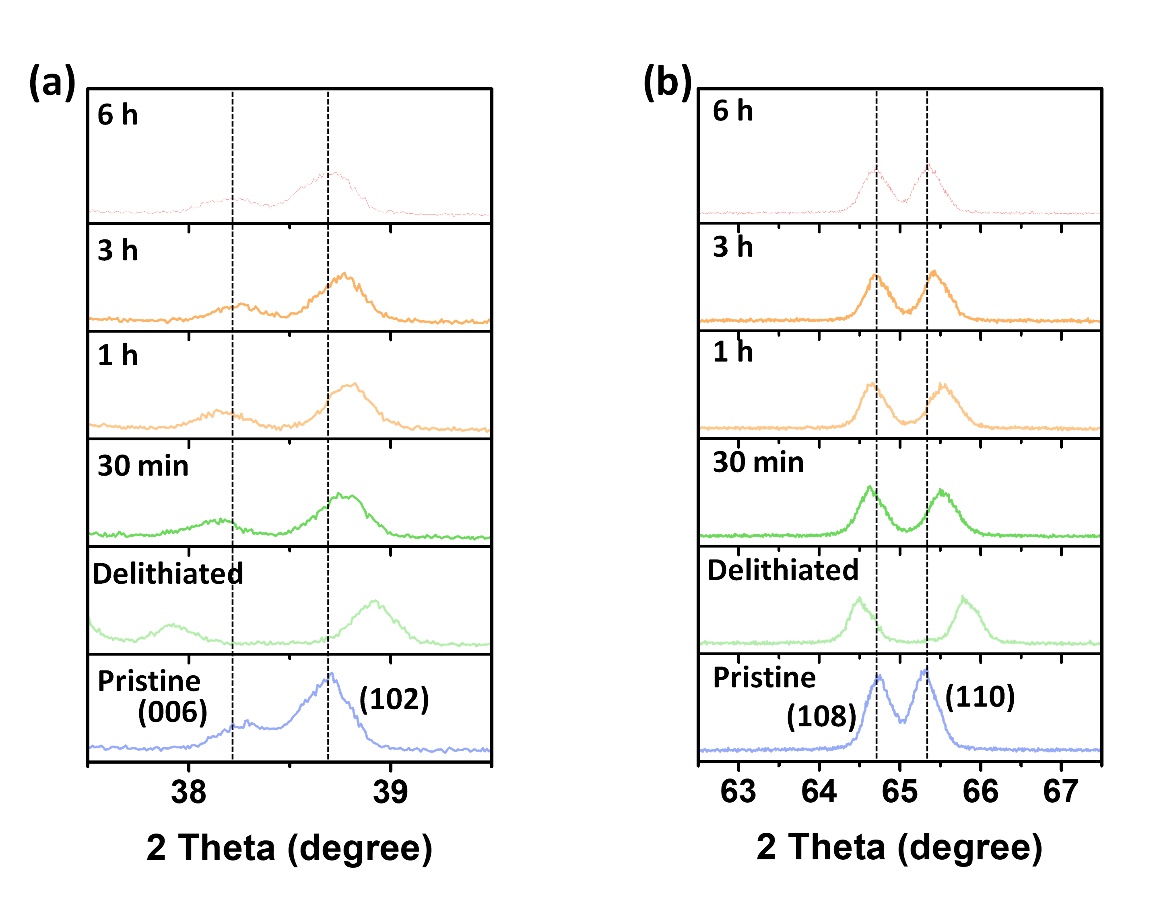


**Figure S4.** *Ex-situ* XRD patterns of the P-NCM, D-NCM, R-NCM (30 min), R-NCM (1 h), R-NCM (3 h), and R-NCM (6 h) (a) Enlargement of the regions in the range of 37.5°–39.5° (c) Enlargement of the regions in the range of 62.5°–67.5°


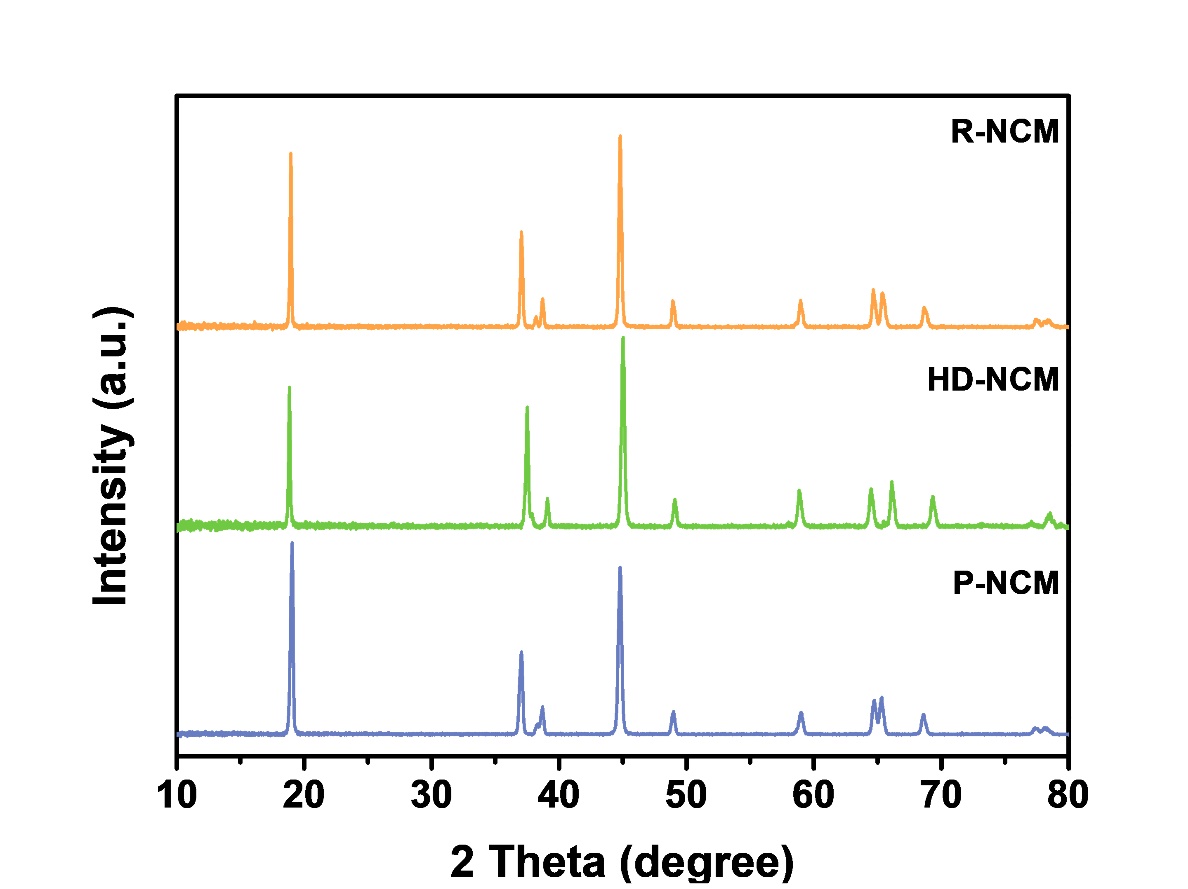


**Figure S5.** *Ex-situ* XRD patterns of the P-NCM, HD-NCM, and R-NCM


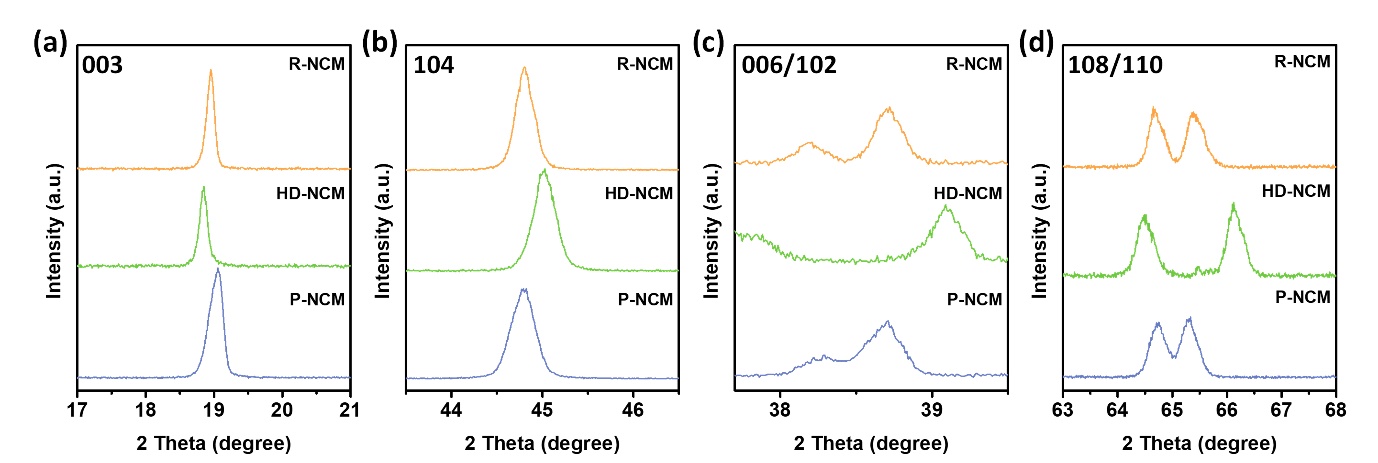


**Figure S6.** (003), (104), (006)/(102), and (108)/(110) *ex-situ* XRD peaks of the P-NCM, HD-NCM, and R-NCM


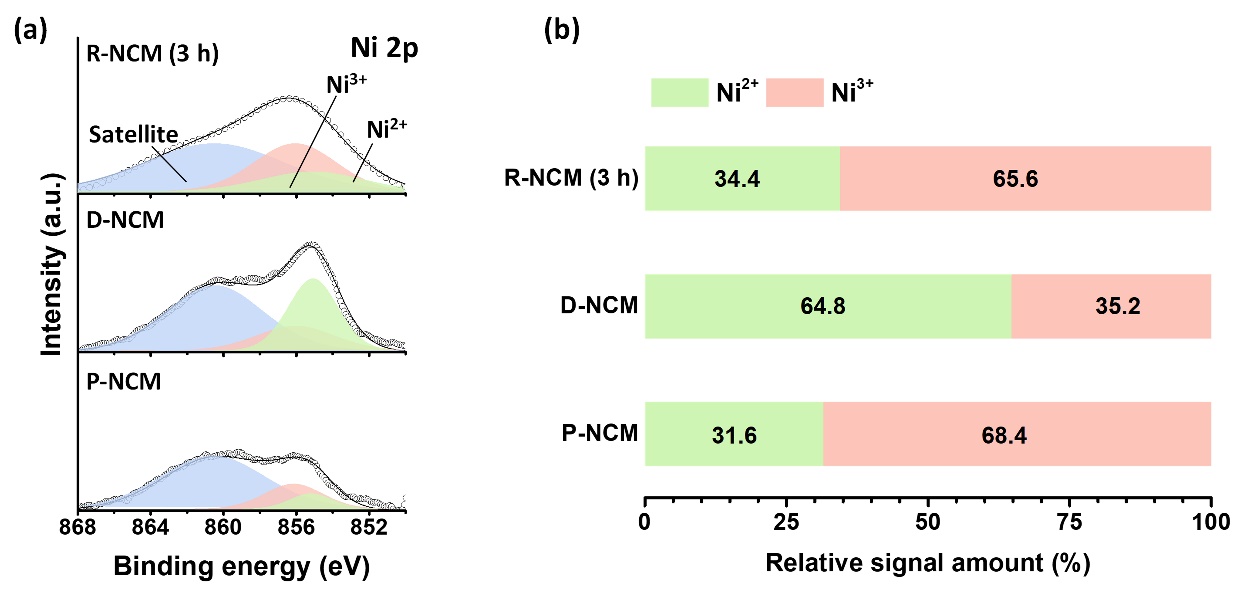


**Figure S7.** (a) *Ex-situ* XPS spectra of the P-NCM, D-NCM, and R-NCM (b) Relative amounts of the Ni^2+^ and Ni^3+^ components in the P-NCM, D-NCM, and R-NCM
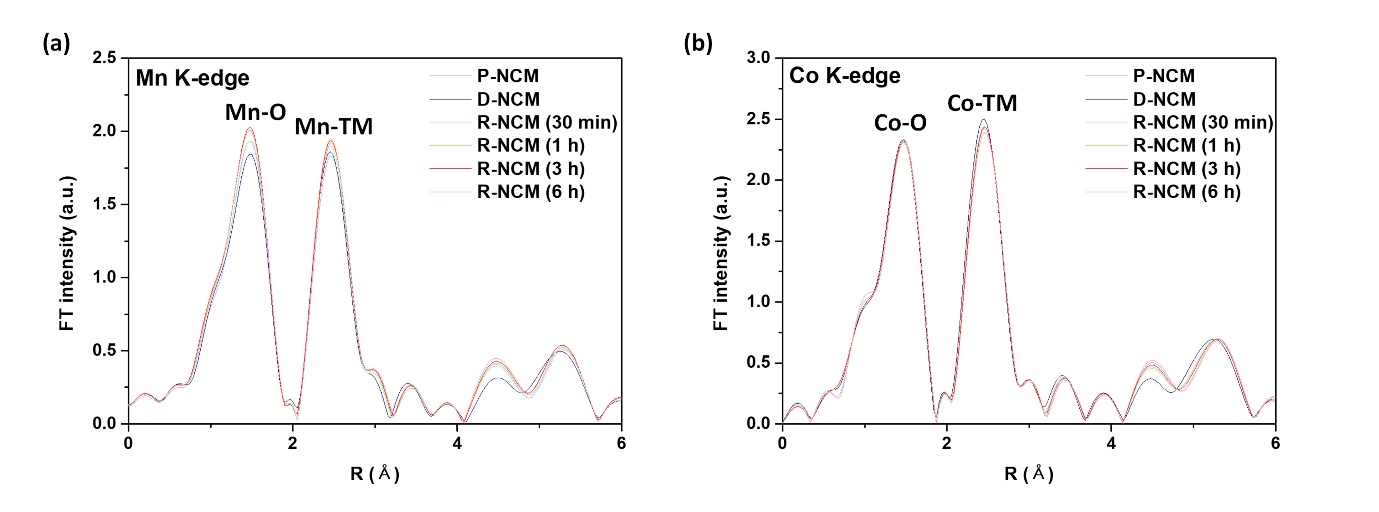


**Figure S8.** Fourier-transformed magnitudes of *k^2^* weighted Mn K-edge and Co K-edge EXAFS spectra


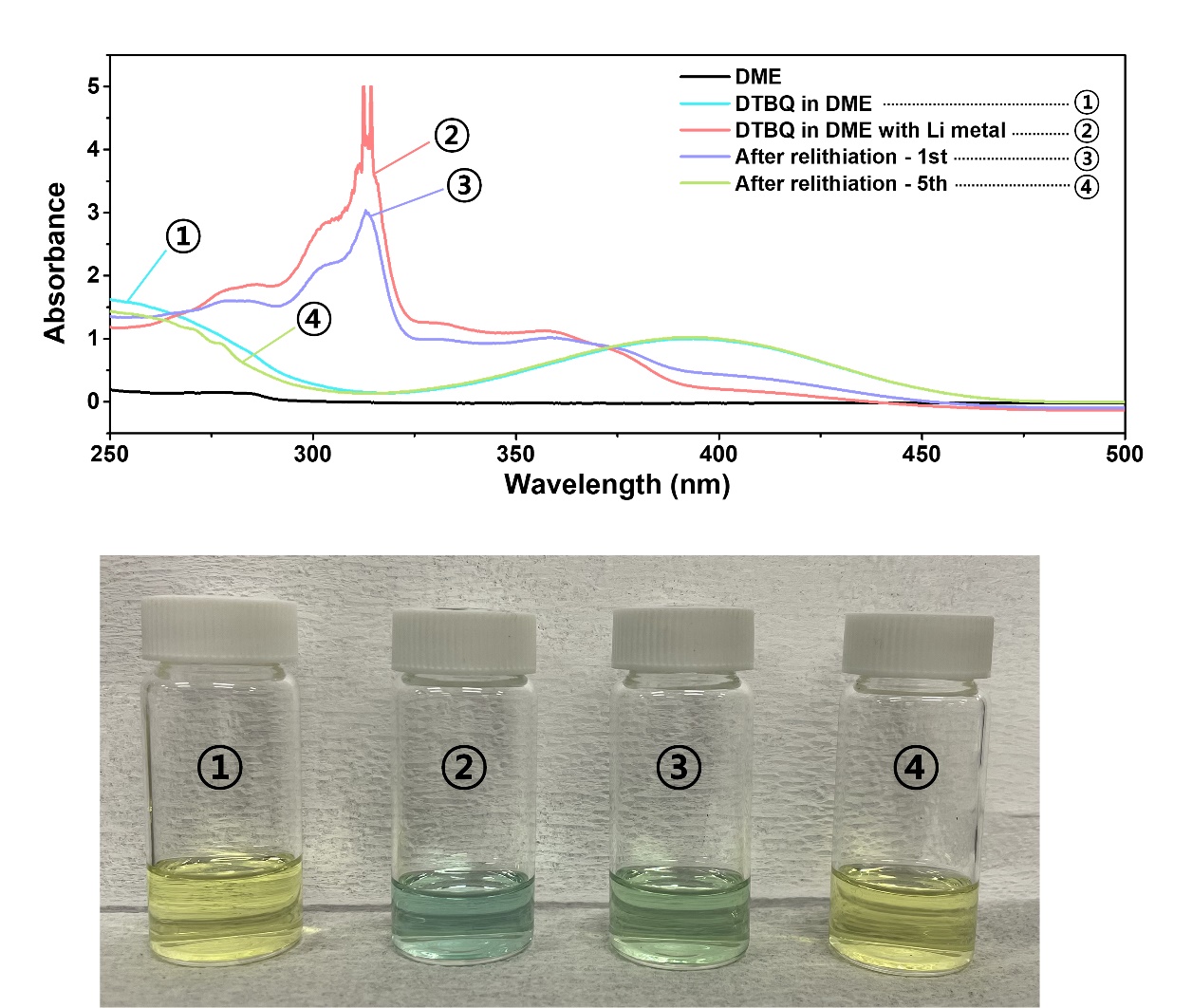


**Figure S9.** UV–vis spectra of DME, DTBQ in DME, Li^+^-coordination DTBQ (contact Li metal with DTBQ in DME), and Li^+^-decoordination DTBQ solutions (after 1^st^ and 5^th^ relithiation). Photographs of the DTBQ in DME, Li^+^-coordination DTBQ, and Li+-decoordination DTBQ (after 1^st^ and 5^th^ relithiation)

**Table S1.** Quantitative structural information for the first Ni-O and Ni-TM bonding in Li_1-x_Ni_0.6_Co_0.2_Mn_0.2_O_2_, obtained by EXAFS curve fitting analysis

| Sample | Path | ^a^ CN | ^b^ σ^2^ [Å^2^] | ^c^ R [Å] | ^e^ R-factor [%] |
| --- | --- | --- | --- | --- | --- |
| P-NCM | Ni-O | 6 | 0.010 | 1.984 | 0.43 |
|  | Ni-TM | 6 | 0.004 | 2.871 |  |
| D-NCM | Ni-O | 6 | 0.006 | 1.890 | 0.36 |
|  | Ni-TM | 6 | 0.005 | 2.827 |  |
| R-NCM (30 min) | Ni-O | 6 | 0.009 | 1.938 | 0.08 |
|  | Ni-TM | 6 | 0.005 | 2.851 |  |
| R-NCM (1 h) | Ni-O | 6 | 0.008 | 1.948 | 0.24 |
|  | Ni-TM | 6 | 0.004 | 2.854 |  |
| R-NCM (3 h) | Ni-O | 6 | 0.009 | 1.952 | 0.06 |
|  | Ni-TM | 6 | 0.005 | 2.857 |  |
| R-NCM (6 h) | Ni-O | 6 | 0.009 | 1.963 | 0.12 |
|  | Ni-TM | 6 | 0.004 | 2.864 |  |

** a: Coordination number, b: Debye–Waller factor, c: Bond length, d: Average bond length, e: EXAFS R-factor*
